# Amyloid-Beta Mediates Homeostatic Synaptic Plasticity

**DOI:** 10.1523/JNEUROSCI.1820-20.2021

**Published:** 2021-06-16

**Authors:** Christos Galanis, Meike Fellenz, Denise Becker, Charlotte Bold, Stefan F. Lichtenthaler, Ulrike C. Müller, Thomas Deller, Andreas Vlachos

**Affiliations:** ^1^Department of Neuroanatomy, Institute of Anatomy and Cell Biology, Faculty of Medicine, University of Freiburg, 79104 Freiburg, Germany; ^2^Faculty of Biology, University of Freiburg, 79104 Freiburg, Germany; ^3^Institute of Clinical Neuroanatomy, Dr. Senckenberg Anatomy, Neuroscience Center, Goethe-University Frankfurt, 60590 Frankfurt, Germany; ^4^Institute of Pharmacy and Molecular Biotechnology, Functional Genomics, Ruprecht-Karls University, 69120 Heidelberg, Germany; ^5^German Center for Neurodegenerative Diseases, 81377 Munich, Germany; ^6^Neuroproteomics, School of Medicine, Klinikum Rechts der Isar, Technical University Munich, 81675 Munich, Germany; ^7^Munich Cluster for Systems Neurology, 81377 Munich, Germany; ^8^Center Brain Links Brain Tools, University of Freiburg, 79110 Freiburg, Germany; ^9^Center for Basics in Neuromodulation, Faculty of Medicine, University of Freiburg, 79106 Freiburg, Germany

**Keywords:** Alzheimer’s disease, amyloid-beta, APP processing, homeostatic plasticity, sAPPalpha, secretases

## Abstract

The physiological role of the amyloid-precursor protein (APP) is insufficiently understood. Recent work has implicated APP in the regulation of synaptic plasticity. Substantial evidence exists for a role of APP and its secreted ectodomain APPsα in Hebbian plasticity. Here, we addressed the relevance of APP in homeostatic synaptic plasticity using organotypic tissue cultures prepared from *APP*^−/−^ mice of both sexes. In the absence of APP, dentate granule cells failed to strengthen their excitatory synapses homeostatically. Homeostatic plasticity is rescued by amyloid-β and not by APPsα, and it is neither observed in *APP^+/+^* tissue treated with β- or γ-secretase inhibitors nor in synaptopodin-deficient cultures lacking the Ca^2+^-dependent molecular machinery of the spine apparatus. Together, these results suggest a role of APP processing via the amyloidogenic pathway in homeostatic synaptic plasticity, representing a function of relevance for brain physiology as well as for brain states associated with increased amyloid-β levels.

## Significance Statement

Considerable effort has been directed to better understand the pathogenic role of the amyloid precursor protein (APP) and its cleavage products in neurodegeneration, with a major focus on the accumulation and deposition of “synaptotoxic” amyloid-β (Aβ) peptides, which are produced by sequential cleavage of APP by β- and γ-secretases. Although the amyloidogenic APP processing pathway has recently been targeted in patients with Alzheimer’s disease, the physiological role of APP/Aβ remains unclear, which limits our understanding of how such interventions could influence brain functions in health and disease. Here, we report an essential role of Aβ (and not APPsα) in homeostatic synaptic plasticity, suggesting that this could be a major physiological function of Aβ in the healthy brain.

## Introduction

In recent years, considerable effort has been directed to better understand the pathogenic role of the amyloid precursor protein (APP) and its cleavage products in neurodegeneration (Bohm et al., 2015). It has been proposed that the accumulation and deposition of “synaptotoxic” amyloid-β (Aβ) peptides, which are produced by sequential cleavage of APP by β- and γ-secretases (Lichtenthaler et al., 2011), are responsible for synapse loss, which is regarded as the significant structural hallmark of cognitive decline in Alzheimer’s disease (AD). In comparison, the physiological functions of cleavage products generated along the “amyloidogenic pathway,” which are also produced in low concentrations in the healthy brain (Seubert et al., 1992; Shoji et al., 1992; Cirrito et al., 2003), are incompletely understood (Kamenetz et al., 2003; Abramov et al., 2009; Puzzo et al., 2011).

Studies using mouse mutants lacking APP or APP-gene family members (Zheng et al., 1995; von Koch et al., 1997; Magara et al., 1999; Herms et al., 2004; Ring et al., 2007) have shed new light on the role of APP in neuronal migration, synaptogenesis, and synaptic structure and function (Müller et al., 2017). Specifically, alterations in dendritic arborization and spine densities have been reported in pyramidal cells of *APP*^−/−^ mice, and these changes are accompanied by defects in the ability of neurons to express long-term potentiation (LTP) of excitatory synaptic strength and alterations in spatial learning (see, e.g., Muller et al., 1994; Zheng et al., 1995; Li et al., 1996; Dawson et al., 1999; Ring et al., 2007; Tyan et al., 2012; for review, see Ludewig and Korte, 2016; Müller et al., 2017). Several of the reported phenotypes of *APP*^−/−^ mice are rescued by the APP secreted ectodomain α (APPsα) (e.g., Weyer et al., 2014; Hick et al., 2015; Richter et al., 2018; Tan et al., 2018). Therefore, it has been proposed that APP could play an important role in neuronal structural and functional plasticity under physiological conditions through the “non-amyloidogenic pathway,” which produces APPsα. The physiological function of Aβ has, however, remained enigmatic.

In an attempt to learn more about the role of APP and its cleavage products in synaptic plasticity, we here tested for its significance in another major plasticity mechanism (i.e., homeostatic synaptic plasticity). The ability of neurons to adjust their synaptic strength in a compensatory manner is considered to be fundamental for physiological brain function (Turrigiano, 1999; Styr and Slutsky, 2018). It is also a relevant compensatory mechanism in the context of brain diseases and a promising therapeutic target (Andre et al., 2018; Smith-Dijak et al., 2019). In contrast to Hebbian synaptic plasticity (e.g., LTP), homeostatic plasticity is based on negative feedback mechanisms (Turrigiano, 2008; Lisman, 2017). Meanwhile, several molecular players have been identified that control homeostatic synaptic plasticity (Stellwagen and Malenka, 2006; Goddard et al., 2007; Cingolani et al., 2008; Sun and Turrigiano, 2011; Walters and Josselyn, 2019). Recently, a potential role of APP in homeostatic synaptic plasticity has been discussed (Hoe et al., 2012; Jang and Chung, 2016; Andre et al., 2018; Styr and Slutsky, 2018). However, the mechanistic link between APP and homeostatic plasticity remains unclear because homeostatic plasticity could be induced by APP and its cleavage products or indirectly by synapse loss and cell death (i.e., denervation/deprivation-induced homeostatic adaptation) (see, e.g., Vlachos et al., 2013; Gilbert et al., 2016; Barnes et al., 2017). Here, we studied the role of APP in non-diseased brain tissue and report an essential role of Aβ in homeostatic plasticity of excitatory neurotransmission, suggesting that this could be one of the major physiological functions of APP/Aβ in the normal brain.

## Materials and Methods

### 

#### 

##### Ethics statement

Mice were maintained in a 12 h light/dark cycle with food and water available *ad libitum*. Every effort was made to minimize the distress and pain of animals. All experimental procedures were performed according to German animal welfare legislation and approved by the appropriate animal welfare committee and the animal welfare officer of Freiburg University.

##### Animals

WT *C57BL/6J*, *SYNPO*^−/−^ (Deller et al., 2003), *APP*^−/−^ (Li et al., 1996), *APPsα-KI* mice (Ring et al., 2007), and their WT littermates were used in this study.

##### Preparation of tissue cultures

Entorhino-hippocampal tissue cultures were prepared at postnatal day 4-5 from mice of either sex as previously described (Vlachos et al., 2012). The incubation medium consisted of 50% (v/v) MEM, 25% (v/v) basal medium eagle (BME), 25% (v/v) heat-inactivated normal horse serum, 25 mm HEPES, 0.15% (w/v) NaHCO_3_, 0.65% (w/v) glucose, 0.1 mg/ml streptomycin, 100 U/ml penicillin, and 2 mm Glutamax. The pH was adjusted to 7.3, and the medium was changed 3 times per week. Tissue cultures were allowed to mature *in vitro* for at least 18 d before any experimental procedure.

##### Whole-cell patch-clamp

Whole-cell patch-clamp recordings of dentate gyrus granule cells were conducted at 35°C. The bath solution contained 126 mm NaCl, 2.5 mm KCl, 26 mm NaHCO_3_, 1.25 mm NaH_2_PO_4_, 2 mm CaCl_2_, 2 mm MgCl_2_, and 10 mm glucose and was saturated with 95% O_2_/5% CO_2_. For miniature and spontaneous AMPAR-mediated excitatory postsynaptic current (mEPSC/sEPSC) recordings as well as current-clamp (input-output) recordings, patch pipettes contained 126 mm K-gluconate, 4 mm KCl, 4 mm ATP-Mg, 0.3 mm GTP-Na_2_, 10 mm PO-creatine, 10 mm HEPES, and 0.1% (w/v) biocytin (pH 7.25 with KOH, 290 mOsm with sucrose). For NMDAR-mediated mEPSC recordings, patch pipettes contained 120 mm CsCH_3_SO_3_, 8 mm CsCl, 1 mm MgCl_2_, 0.4 mm EGTA, 2 mm ATP-Mg, 0.3 mm GTP-Na_2_, 10 mm PO-creatine, 10 mm HEPES, and 5 mm QX-314 (pH 7.25 with CsOH, 295 mOsm with sucrose). For miniature and spontaneous inhibitory postsynaptic current (mIPSC/sIPSC) recordings, patch pipettes contained 40 mm CsCl, 90 mm K-gluconate, 1.8 mm NaCl, 1.7 mm MgCl_2_, 3.5 mm KCl, 0.05 mm EGTA, 2 mm ATP-Mg, 0.4 mm GTP-Na_2_, 10 mm PO-creatine, 10 mm HEPES (pH 7.25, with CsOH, 270 mOsm with sucrose). AMPAR-mediated mEPSCs were recorded in the presence of 10 μm D-APV and 0.5 μm TTX, NMDAR-mediated mEPSCs in the presence of 10 μm CNQX and 0.5 μm TTX, and mIPSCs in the presence of 0.5 μm TTX, 10 μm D-APV, and 10 μm CNQX. sEPSCs were recorded without the addition of any drugs in the bath solution and sIPSCs in the presence of 10 μm D-APV and 10 μm CNQX. Current-clamp recordings were performed in the presence of 10 μm D-APV, 10 μm CNQX, and 10 μm bicuculline methiodide. Neurons were recorded at a holding potential of −70 mV for mIPSCs/sIPSCs and AMPAR-mediated mEPSCs/sEPSCs. NMDAR-mediated mEPSCs were acquired at +40 mV. For current-clamp recordings, neurons were hyperpolarized with −100 pA and then depolarized up to +200 pA with 1-s-long 10 pA current injection steps. Series resistance was monitored in 2 min intervals and recordings were discarded if the series resistance reached ≥30 MΩ and the leak current changed significantly.

##### Reconstruction of dendritic trees and spine density analysis

Dentate granule cells were patched with Alexa-568 (10 μm) added to the internal solution and filled for 10 min in whole-cell configuration for visualization of identified cells. Confocal image stacks of granule cells (512 × 512 pixel, voxel size 0.49 × 0.49 × 2 µm) were acquired directly at the electrophysiology setup using a Zeiss LSM Exciter confocal microscope with a 40× water immersion objective lens (0.8 NA; Carl Zeiss). Granule cells were manually reconstructed in 3D and analyzed using Neurolucida/NeuroExplorer software (MBF Bioscience). Total dendritic length (TDL) was calculated as the sum of length of all reconstructed dendritic segments of a given cell. For Sholl analysis, concentric spheres with diameters increasing in 20 µm increments were drawn around the cell soma, and the number of dendrites intersecting each sphere was calculated.

##### Immunostaining and imaging

Tissue cultures were fixed in a solution of 4% (w/v) paraformaldehyde (PFA) and 4% (w/v) sucrose in 0.01 m PBS for 1 h, followed by 2% (w/v) PFA and 30% (w/v) sucrose in 0.01 m PBS overnight. For synaptopodin (Synpo) staining, 30 μm cryosections were prepared and stained with antibodies against synaptopodin (1:1000; SE-19 Sigma Millipore, RRID:AB_261570). Sections were incubated for 1 h with 10% (v/v) normal goat serum (NGS) in 0.5% (v/v) Triton X-100-containing PBS to reduce unspecific antibody binding and incubated for 24 h at 4°C with the primary antibody in PBS with 10% NGS and 0.1% Triton X-100. Sections were washed and incubated for 4 h with appropriate Alexa-labeled secondary antibodies (Invitrogen; 1:1000, in PBS with 10% NGS, 0.1% Triton X-100). DAPI nuclear stain was used to visualize cytoarchitecture (1:5000; in 0.01 m PBS for 15 min). Sections were washed with 0.01 m PBS, transferred onto glass slides, and mounted for visualization with anti-fading mounting medium. Confocal images were acquired using a Leica Microsystems TCS SP8 laser scanning microscope with 20× (NA 0.75), 40× (NA 1.30), and 63× (NA 1.40) oil-submersion objectives.

##### *Post hoc* identification of recorded neurons

Tissue cultures were fixed in a solution of 4% (w/v) PFA and 4% (w/v) sucrose in 0.01 m PBS for 1 h. The fixed tissue was incubated for 1 h with 10% (v/v) NGS and 0.5% (v/v) Triton X-100 in 0.01 m PBS. Biocytin (Sigma Millipore, catalog #B4261) filled cells were stained with Alexa-488-, Alexa-568-, or Alexa-633-conjugated streptavidin (Thermo Fisher Scientific; 1:1000; in 0.01 m PBS with 10% NGS and 0.1% Triton X-100) for 4 h, and DAPI (Thermo Fisher Scientific) staining was used to visualize cytoarchitecture (1:5000; in 0.01 m PBS for 15 min). Slices were washed, transferred, and mounted onto glass slides for visualization. Transfected and streptavidin-stained granule cells were visualized with a Leica Microsystems TCS SP8 laser scanning microscope with 20× (NA 0.75), 40× (NA 1.30), and 63× (NA 1.40) oil-submersion objectives. Outer molecular layer segments were imaged with higher scan zoom, and spine densities were determined as described previously (Hick et al., 2015).

##### Electron microscopy

*APP^+/+^* and *APP^−/−^* tissue cultures were fixed in 4% (w/v) PFA and 2% (w/v) glutaraldehyde in 0.1 m PB overnight and washed for 1 h in 0.1 m PB. After fixation, tissue cultures were sliced with a vibratome, and the 50 μm slices were incubated with 1% (w/v) osmium tetroxide for 20 min in 5% (w/v) sucrose containing 0.1 m PB. The slices were washed 5 times for 10 min in 0.1 m PB and washed in graded ethanol [10 min in 10% (v/v) and 10 min in 20 (v/v)]. The slices were then incubated with uranyl acetate [1% (w/v) in 70% (v/v) ethanol)] overnight and were subsequently dehydrated in graded ethanol 80% (v/v), 90% (v/v), and 98% (v/v) for 10 min. Finally, slices were incubated with 100% (v/v) ethanol 2 times for 15 min followed by two 15 min washes with propylene oxide. The slices were then transferred for 30 min in a 1:1 mixture of propylene oxide with durcupan and then for 1 h in durcupan. The durcupan was exchanged with fresh durcupan, and the slices were transferred in 4°C overnight. The slices were then embedded between liquid release-coated slides and coverslips. Cultures were re-embedded in blocks, and ultrathin sections were collected on copper grids (compare Radic et al., 2017; Lenz et al., 2021). Electron microscopy was performed at a LEO 906E microscope (Carl Zeiss) at 4646× magnification. Acquired images were saved as TIF files and analyzed using the ImageSP Viewer software (http://sys-prog.com). For each tissue culture, 10 images in the outer molecular layer of the dentate gyrus were analyzed. Dendritic spines were identified, and the sizes of postsynaptic densities (PSDs) and dense plates of spine apparatuses were assessed with the line tool.

##### Pharmacology

Tissue cultures were treated with TTX (2 μm; BioTrend, catalog #BN0518), D-APV (50 μm; Tocris, catalog #0106), synthetic Αβ_1-42_ (1.5 μm; Bachem, catalog #4014447) or Aβ_42-1_ (1.5 μΜ; Bachem, catalog #H-3976) (Novotny et al., 2016), synthetic Αβ_1-40_ (1.5 μm; Bachem, catalog #4035886) recombinant APPsα (10 nm) (Hick et al., 2015); β-site APP cleaving enzyme (BACE) inhibitor C3 (20 μm; Calbiochem, Sigma Millipore, catalog #565788), Begacestat (1 μm; Tocris, catalog #4283), KN-92 (5 μm; Tocris, catalog #4130), KN-93 (5 μm; Tocris, catalog #1278), BNT77 (1.3 μg/ml; Wako, catalog #014-26881, RRID:AB_2827702), JRD32 (1.3 μg/ml; RRID:AB_2827703) (Hick et al., 2015) for 2 d. For the Aβ treatment, 0.01 m PBS was added to the lyophilized Aβ peptides, followed by 3 × 1 min vortexing on wet ice, and stocks were kept at −80°C. Before treating the tissue cultures, Aβ_1-40_, Aβ_1-42_, and Aβ_42-1_ were slowly thawed on ice and vortexed again before adding to the medium.

##### Viral transduction

Tissue cultures were transfected between 3 and 4 days in vitro (DIV) by adding 1 μl of AAV-Syn/Venus, AAV-Syn/Venus_T2A_APPsα (AAV-APPsα) or 1 μl of AAV-Syn/APP695_T2A_Venus (AAV-flAPP-Venus; kindly provided by Drs. Christian Buchholz and Tobias Abel) directly on top of each culture. Tissue cultures were then left to mature at least until 18 DIV before experimental assessment.

##### ELISA assay and Aβ peptide quantification

Incubation medium from vehicle-only (control) and pharmacologically treated 3-week-old tissue cultures was collected, frozen immediately with dry ice, and stored at −80°C (vehicle, *n* = 6 wells with 36 tissue cultures; BACE inhibitor C3, *n* = 3 wells with 18 tissue cultures; Begacestat, *n* = 3 wells with 18 tissue cultures). For the detection of Aβ, a V-PLEX Aβ Peptide Panel 1 Kit (MesoScale Discovery, catalog #K15199E, RRID:AB_2827747) was used with a ruthenylated anti-Αβ antibody (4G8 clone). The Aβ peptide concentration was determined using the MSD Discovery Workbench software (MesoScale Discovery).

##### Conditioned concentrated incubation medium

Incubation medium from two wells in which *APP^+/+^* tissue cultures were cultivated was collected and concentrated in an Amicon Ultra-2 Centrifugal Filter Unit with a cut off molecular weight of 10 kDa (Millipore, catalog #UFC201024). Media were centrifuged for 20 min at 4000 × *g* at room temperature. The permeate (containing proteins < 10 kDa) was collected, and the retentate (with concentrated proteins ≥ 10 kDa) was recovered from the column by a second centrifugation at 1000 × *g* for 2 min. The permeate and the retentate were added to mature (≥18 DIV) *APP^−/−^* tissue cultures. *APP^−/−^* preparations were cultivated in a mix of 50% permeate and 50% standard incubation medium since the permeate was free of large serum proteins. The concentrated retentate (final volume 80–90 μl) was used with 900 μl of the standard incubation medium.

##### Experimental design and statistical analysis

Analyses were performed with the person analyzing the data blind to the experimental condition. One or two tissue cultures were used from each animal (numbers of tissue cultures used per experimental group is denoted in the figure legends). Electrophysiological data were analyzed using pClamp 10.7 software suite (Molecular Devices), MiniAnalysis (Synaptosoft), and Igor Pro 7 (Wavemetrics). Up to 300 events were analyzed per recorded neuron. Sizes of immunolabeled synaptopodin clusters were assessed using the FIJI ImageJ software package (available from https://imagej.net/ImageJ) as described previously (Vlachos et al., 2013). Numbers and sizes of spine apparatus and PSDs were calculated using the ImageSP Viewer software (http://sys-prog.com). Statistical comparisons were made using Mann–Whitney test (to compare two groups), Wilcoxon test (to compare recordings made from the same cells before and after treatment), Kruskal–Wallis test followed by Dunn’s *post hoc* test for multiple group testing or one or two-way ANOVA as indicated in the figure captions and text (GraphPad Prism 7, GraphPad Software). *p* values of <0.05 were considered a significant difference. All values represent mean ± SEM.

##### Digital illustrations

Confocal image stacks were exported as 2D projections and stored as TIF files. Figures were prepared using Photoshop graphics software (Adobe). Image brightness and contrast were adjusted.

## Results

### Homeostatic synaptic plasticity is not observed in dentate granule cells of APP-deficient entorhinal-hippocampal tissue cultures

**Figure 1.**
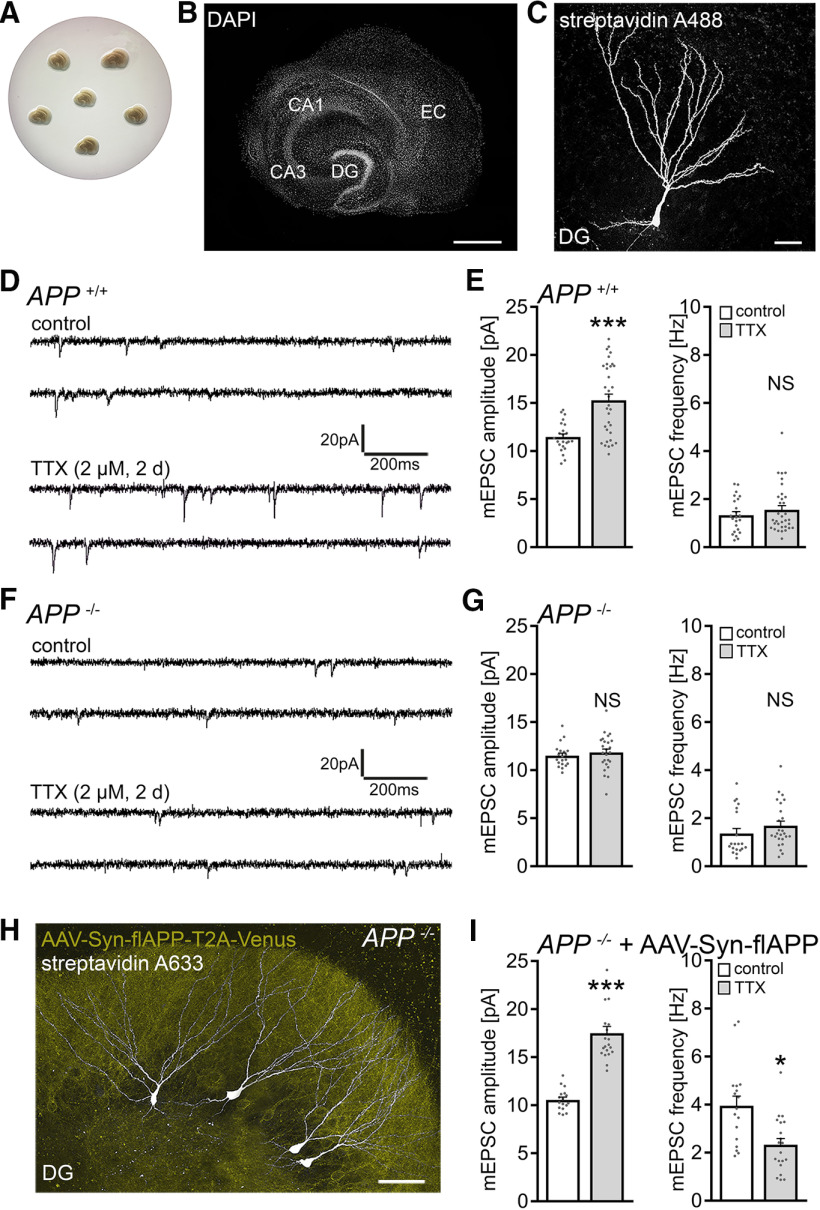
Dentate granule cells of APP-deficient entorhinal-hippocampal tissue cultures do not express homeostatic synaptic plasticity. ***A***, ***B***, Example of 3-week-old entorhinal-hippocampal tissue cultures on a membrane insert and overview at higher magnification of a representative culture. DAPI nuclear staining was used for visualization of cytoarchitecture. DG, Dentate gyrus; EC, entorhinal cortex; CA1 and CA3, cornu ammonis areas 1 and 3. Scale bar, 500 μm. ***C***, Patched dentate granule cell filled with biocytin and identified *post hoc* with streptavidin A488. Scale bar, 25 μm. ***D***, ***E***, Sample traces and group data of AMPAR-mediated mEPSCs recorded from granule cells in vehicle-treated (control) and TTX-treated *APP^+/+^* cultures (control, *n* = 23 cells from 6 cultures; TTX, *n* = 33 cells from 8 cultures; Mann–Whitney test). ***F***, ***G***, Sample traces and group data of AMPAR-mediated mEPSCs recorded from APP-deficient (*APP*^−/−^) dentate granule cells (control, *n* = 20 cells from 6 cultures; TTX, *n* = 25 cells from 7 cultures; Mann–Whitney test). ***H***, Example of *post hoc* identified recorded dentate granule cells (streptavidin 633, white) in an *APP*^−/−^ tissue culture transduced with AAV vectors expressing full-length *APP* (AAV-Syn-flAPP-T2A-Venus; yellow). Scale bar, 50 μm. ***I***, Expression of the flAPP rescues the ability of dentate granule cells in *APP*^−/−^ tissue cultures to express TTX-induced homeostatic synaptic plasticity (control, *n* = 17 cells from 6 cultures; TTX, *n* = 20 cells from 7 cultures; Mann–Whitney test). For mEPSC amplitude, one data point is outside the axis limits in the TTX group. Individual data points are indicated in this and the following figures by gray dots. Data are mean ± SEM. NS, Not significant. *p < 0.05. ***p < 0.001.

Considering the role of the hippocampal formation and specifically the dentate gyrus in memory formation (Friedman and Goldman-Rakic, 1988; Aimone et al., 2011), 3-week-old (≥18 DIV) organotypic tissue cultures containing the entorhinal cortex and the hippocampus were prepared from *APP^+/+^*and *APP*^−/−^ mice, including age- and time-matched *APP*^+/+^ littermates obtained from *APP*^+/−^ intercrossing (Fig. 1*A*,*B*). Tissue cultures were treated with TTX (2 μm; 2 d) to block network activity, and AMPAR-mediated mEPSCs were recorded from individual dentate granule cells (Fig. 1*C*) to assess compensatory (i.e., homeostatic) synaptic changes.

In line with previous work (e.g., Turrigiano et al., 1998; Echegoyen et al., 2007; Kim and Tsien, 2008; Vlachos et al., 2013; Strehl et al., 2018), a homeostatic increase in excitatory synaptic strength (i.e., a robust increase in mEPSC amplitudes) was observed in the WT tissue cultures (Fig. 1*D*,*E*). In *APP*^−/−^ preparations, no significant changes in mEPSC properties were observed in dentate granule cells (Fig. 1*F*,*G*). Specifically, mean mEPSC amplitude was 11.5 ± 0.3 pA in vehicle-only-treated and 11.8 ± 0.4 pA TTX-treated *APP*^−/−^ dentate granule cells (*p* = 0.4; Mann–Whitney test).

In an attempt to rescue the ability of granule cells to express homeostatic synaptic plasticity, *APP*^−/−^ tissue cultures were transfected with a bicistronic AAV vector expressing full-length murine APP (flAPP) and membrane-anchored Venus linked by a T2A site under the control of the neuronal synapsin promoter (AAV-Syn-flAPP-T2A-Venus; Fig. 1*H*). Cultures were transduced at 4-5 DIV and allowed to mature for at least 18 DIV before experimental assessment. A significant compensatory increase in mEPSC amplitudes from 10.5 ± 0.3 pA to 17.5 ± 0.7 pA (*p* < 0.001; Mann–Whitney test; Fig. 1*I*) was observed in the TTX group, while increased mEPSC frequencies were observed in the untreated cultures that were reduced after TTX treatment (Fig. 1*I*). Increased mEPSC frequencies under control conditions were attributed to viral transduction in these experiments since transfections of APP-deficient preparations with AAV-Syn-Venus resulted in increased mEPSC frequencies without affecting mEPSC amplitudes (amplitude: control, 11.7 ± 0.3 pA; transfected, 11.8 ± 0.4 pA; Mann–Whitney test; *p* = 0.74; frequency: control, 2.5 ± 0.2 Hz; transfected, 6.6 ± 0.7 Hz; Mann–Whitney test; *p* < 0.001; *n* = 12 cells from 4 cultures in each group). We conclude from these results that postnatal expression of APP is required for TTX-induced homeostatic scaling of excitatory synapses to occur in cultured dentate granule cells.

**Figure 2.**
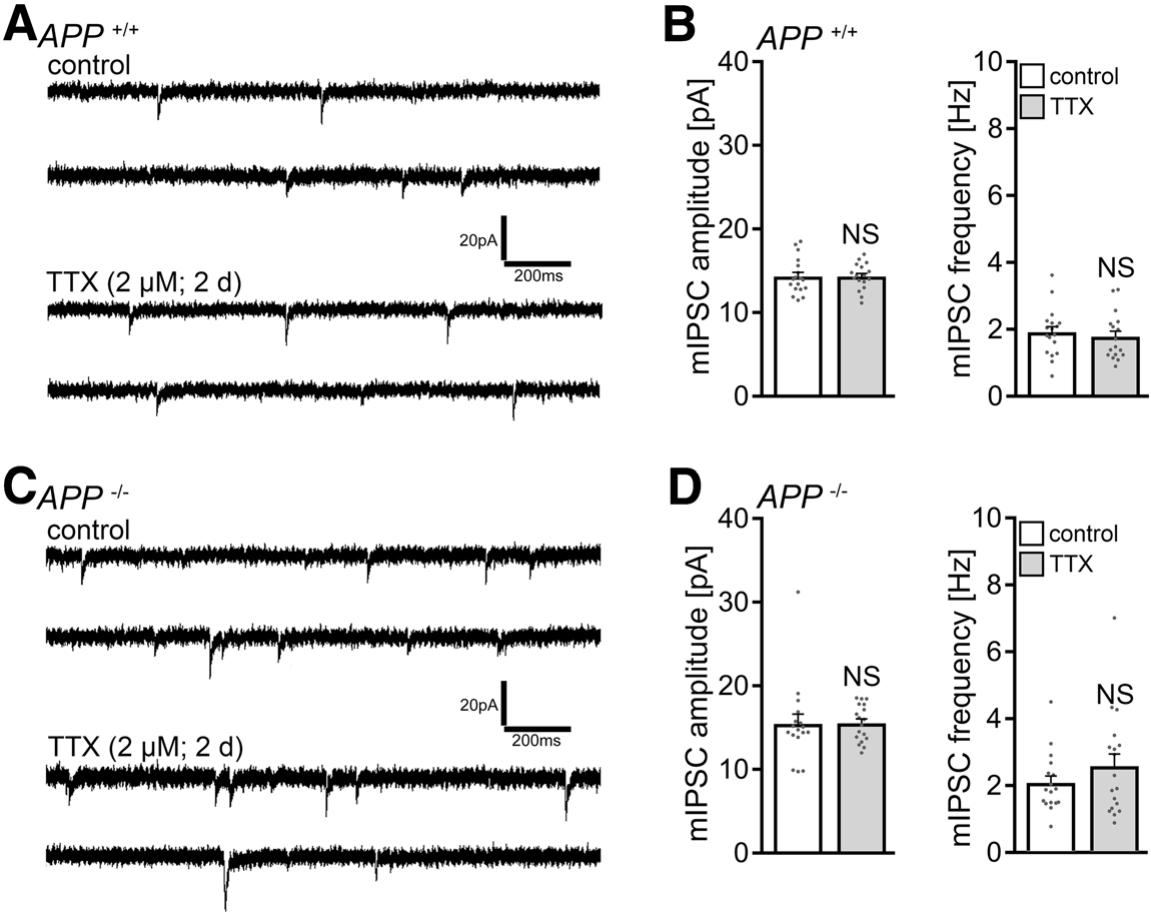
Dentate granule cells of WT and of APP-deficient tissue cultures do not adjust their inhibitory synapses following TTX treatment. ***A***, ***B***, Sample traces and group data of mIPSCs recorded from granule cells in vehicle-only-treated (control) and TTX-treated *APP^+/+^* cultures (control, *n* = 18 cells from 6 cultures; TTX, *n* = 17 cells from 6 cultures; Mann–Whitney test). ***C***, ***D***, Sample traces and group data of mIPSCs recorded in vehicle-only-treated (control) and TTX-treated *APP^−/−^* dentate granule cells (control, *n* = 17 cells from 6 cultures; TTX, *n* = 18 cells from 6 cultures; Mann–Whitney test). Data are mean ± SEM. NS, Not significant.

Based on our recent work (Lenz et al., 2019), we also tested for changes in inhibitory synaptic strength, and we did not detect TTX-induced changes in mIPSCs of dentate granule cells, either in *APP^+/+^* or *APP*^−/−^ tissue cultures (Fig. 2). Hence, we focused on the role of APP in excitatory synaptic scaling.

### No significant alterations in basic functional and structural properties of APP-deficient dentate granule cells

**Figure 3.**
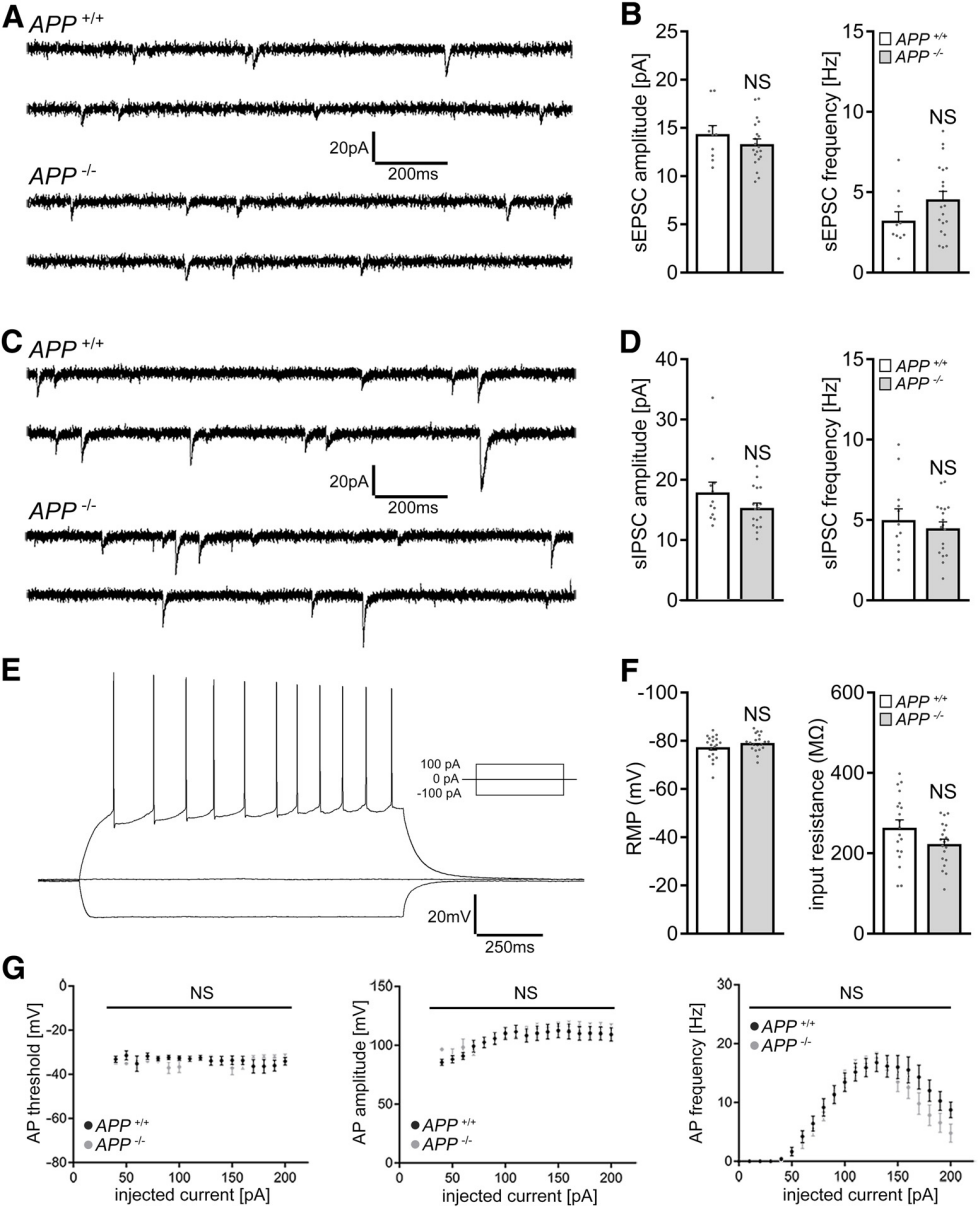
APP deficiency does not affect basic functional properties of cultured dentate granule cells. ***A***, ***B***, Sample traces and group data of sEPSCs recorded from dentate granule cells of *APP^+/+^* and *APP*^−/−^ tissue cultures (*APP*^+/+^, *n* = 10 cells from 4 cultures; *APP*^−/−^, *n* = 20 cells from 6 cultures; Mann–Whitney test). ***C***, ***D***, Sample traces and group data of sIPSCs from granule cells of *APP^+/+^* and *APP*^−/−^ tissue cultures (*APP^+/+^*, *n* = 12 cells from 4 cultures; *APP*^−/−^, *n* = 18 cells from 6 cultures; Mann–Whitney test). ***E–G***, Sample traces and group data for input-output properties of dentate granule cells of *APP^+/+^* and *APP*^−/−^ tissue cultures. RMP, Resting membrane potential; AP, action potential. *APP^+/+^*, *n* = 19 cells from 5 cultures; *APP*^−/−^, *n* = 20 cells from 5 cultures (Mann–Whitney test and two-way ANOVA). Data are mean ± SEM. NS, Not significant.

To test whether alterations in baseline synaptic activity explain the inability of *APP*^−/−^ granule cells to express homeostatic excitatory synaptic plasticity, sEPSCs and sIPSCs were recorded in a different set of 3-week-old tissue cultures (Fig. 3*A–D*). No significant differences between the two genotypes were observed in these experiments. Similarly, the input-output properties of dentate granule cells (Fig. 3*E*,*F*), as well as basic properties of action potentials (Fig. 3*G*), were not significantly different between the two groups.

**Figure 4.**
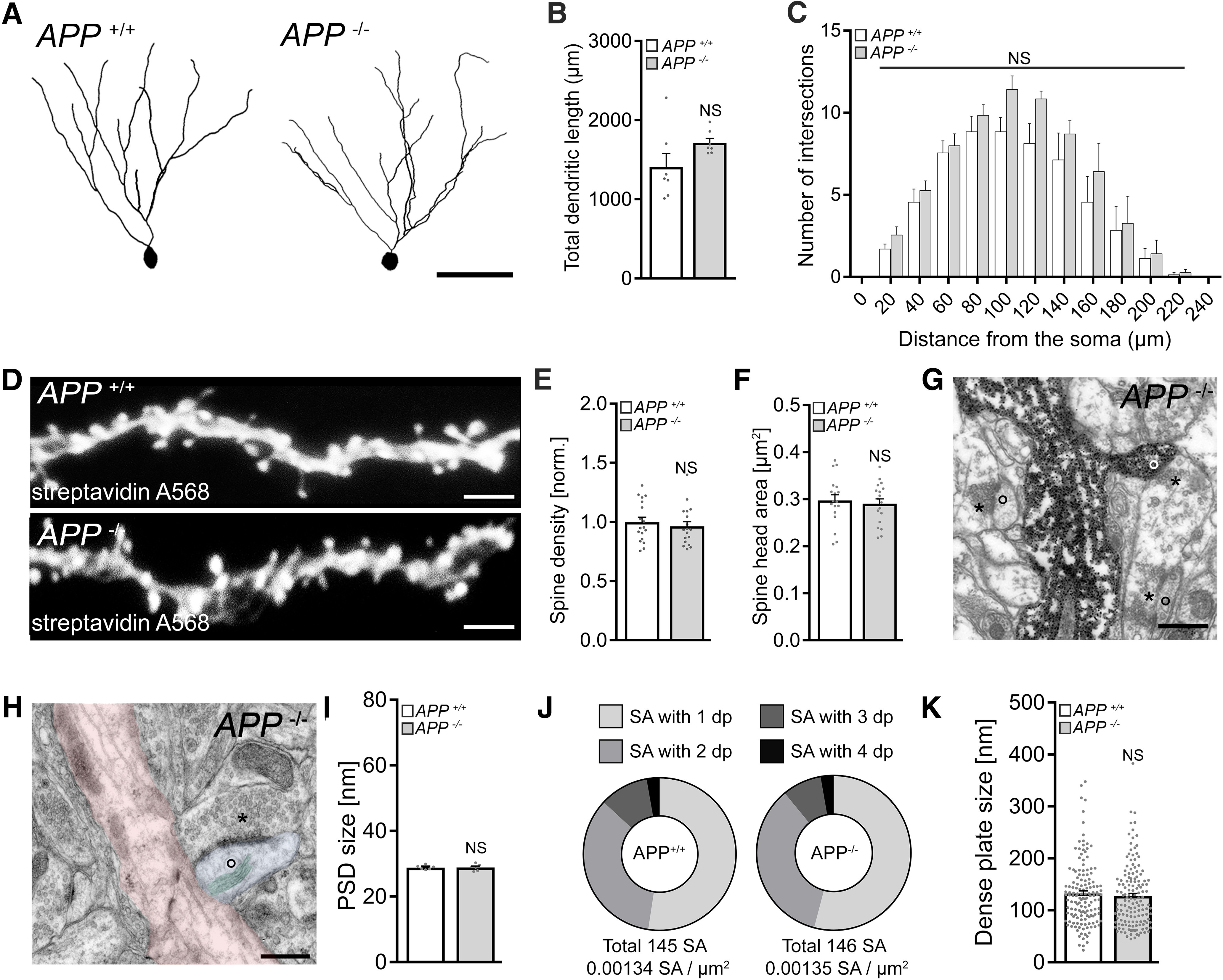
No significant structural differences are observed in granule cells of APP-deficient preparations. ***A-C***, Examples and group data of total dendritic length and Sholl sphere analysis of dentate granule cells in *APP^+/+^* and *APP*^−/−^ tissue cultures (*n* = 7 cells from 7 cultures per group; Mann–Whitney test and two-way ANOVA). Scale bar, 50 μm. ***D–F***, Spine densities and spine head sizes of dendritic segments in the outer molecular layer (oml). Individual data points indicate mean values per dendritic segment; ***E***, *n* = 18 segments from 4 tissue cultures per group; ***F***, Mean spine head size per dendritic segment; *n* = 18 dendritic segments from 4 tissue cultures per group; Mann–Whitney test. Scale bar, 5 µm. ***G***, ***H***, Electron micrograph of synaptic contacts of *APP*^−/−^ dentate granule cells in the oml. Asterisks represent examples of presynaptic compartments. Circles represent postsynapses. Scale bar, 0.5 µm. ***H***, Example of a spine apparatus organelle (SA, green overlay) in a dendritic spine (blue overlay) in the oml of *APP^−/−^* tissue (parent dendrite, red), Scale bar, 0.5 µm. ***I***, Group data of PSD sizes in the oml (*n* = 6 tissue cultures per group; Mann–Whitney test). ***J***, ***K***, Analysis of SA cross sections in the oml of *APP^+/+^* and *APP^−/−^* tissue cultures. Group data for number of SAs/µm^2^, number of dense plates (dp), and the dp sizes in cross sections of electron micrographs (*APP^+/+^*, *n* = 145 SA from 6 tissue cultures, total area analyzed 108,251 μm^2^; *APP^−/−^*, *n* = 146 SA from 6 tissue cultures, total area analyzed 108,159 μm^2^; Mann–Whitney test). Data are mean ± SEM. NS, Not significant.

We also tested for variations in basic structural properties of granule cells between *APP^+/+^* and *APP*^−/−^ preparations. As shown in Figure 4, an assessment of total dendritic branch length and Sholl analysis revealed no significant differences between the genotypes (Fig. 4*A–C*). We also did not find any significant differences in spine numbers and sizes (Fig. 4*D–F*), and synapses were regularly observed in electron microscopy cross-sections of recorded and *post hoc* stained *APP*^−/−^ dentate granule cells (Fig. 4*G*). Consistent with our electrophysiological recordings, ultrastructural assessment of postsynaptic densities in the molecular layer of the dentate gyrus did not show any significant differences between the genotypes (Fig. 4*H*,*I*). Likewise, no significant differences in the number and sizes of spine apparatus organelles (Gray, 1959; Spacek, 1985; Spacek and Harris, 1997), which have been linked to the ability of neurons to express synaptic plasticity (Deller et al., 2003; Vlachos et al., 2012; Lenz et al., 2021), were observed between *APP^−/−^* and *APP^+/+^* preparations (Fig. 4*J*,*K*). Hence, basic functional and (ultra)structural alterations do not readily explain the inability of *APP*^−/−^ granule cells to scale their excitatory synapses.

### A secreted factor rescues the ability of APP-deficient dentate granule cells to express homeostatic synaptic plasticity

In our viral transduction experiments, in which we used flAPP to rescue homeostatic synaptic plasticity of *APP*^−/−^ granule cells (compare Fig. 1*H*,*I*), we noticed that granule cells not expressing flAPP also showed increased mEPSC amplitudes after TTX treatment. Thus, we hypothesized that a secreted factor, and not flAPP expression per se, mediates the effects of APP on TTX-induced homeostatic synaptic plasticity.

**Figure 5.**
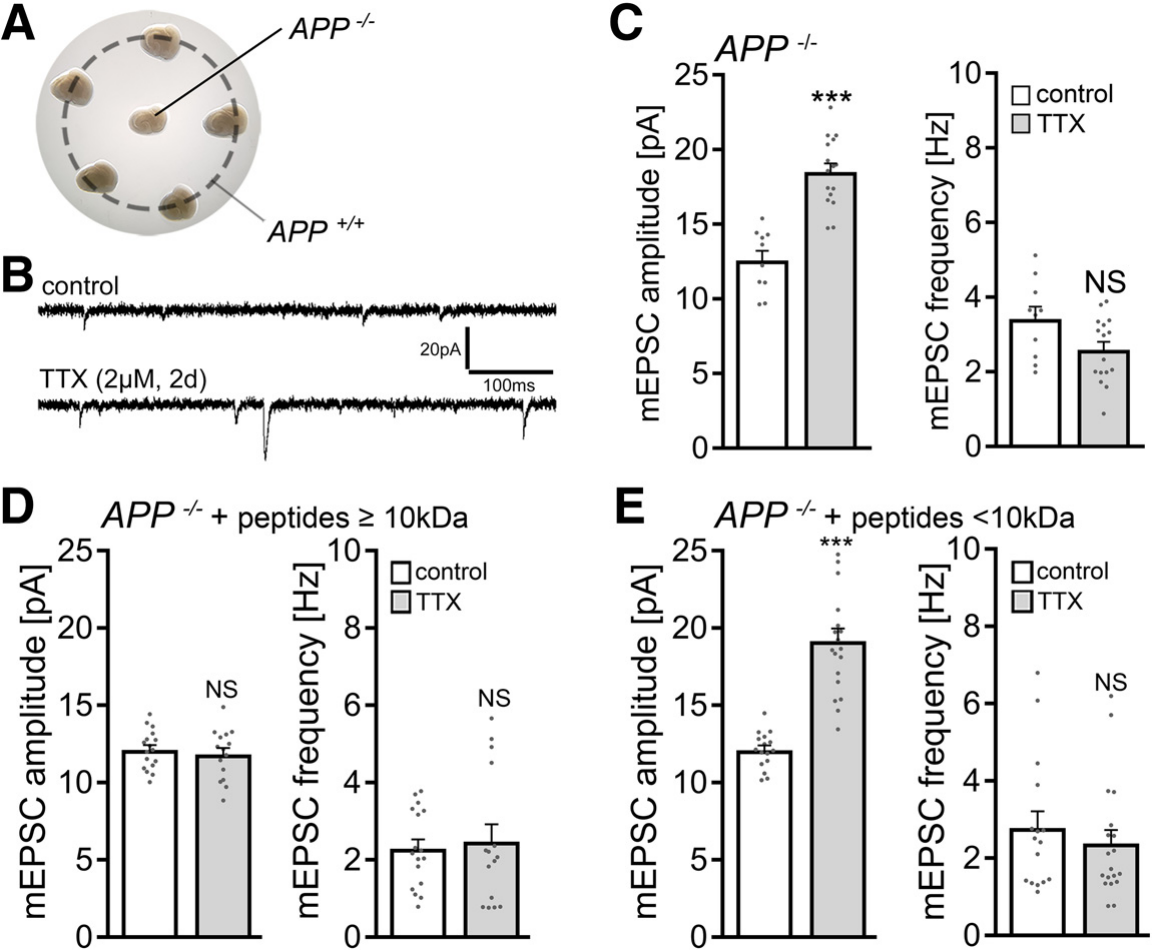
A secreted factor mediates the ability of *APP^−/−^* dentate gyrus granule cells to express homeostatic synaptic plasticity. ***A***, Example of co-cultured *APP^+/+^* and *APP*^−/−^ tissue preparations on the same membrane insert. ***B***, ***C***, Sample traces and group data of AMPAR-mediated mEPSCs recorded from dentate gyrus granule cells in vehicle-treated (control) and TTX-treated (2 μm, 2 d) *APP*^−/−^ tissue cultures (control, *n* = 10 cells from 4 cultures; TTX, *n* = 16 cells from 5 cultures; Mann–Whitney test). ***D***, ***E***, Group data of AMPAR-mediated mEPSCs recorded from granule cells in vehicle-treated (control) and TTX-treated (2 μm, 2 d) *APP*^−/−^ tissue cultures in the presence of WT conditioned medium containing proteins larger (***D***) and smaller (***E***) than 10 kDa (***D***: control, *n* = 17 cells from 5 cultures; TTX, *n* = 15 cells from 4 cultures; ***E***: control, *n* = 16 cells from 4 cultures; TTX, *n* = 19 cells from 4 cultures; Mann–Whitney test). For mEPSC amplitude, one data point is outside the axis limits in the TTX group. Data are mean ± SEM. NS, Not significant. ***p < 0.001.

To test this hypothesis, *APP*^−/−^ tissue was cultured with 5 *APP^+/+^* cultures on the same membrane insert as shown in Figure 5*A*. In these experiments, a significant increase in excitatory synaptic strength was detected in dentate granule cells of TTX-treated *APP*^−/−^ cultures (Fig. 5*B*,*C*). In an independent round of experiments, culturing medium collected from *APP^+/+^* cultures was concentrated to obtain peptide fractions at a cutoff molecular weight of 10 kDa. The medium containing peptides <10 kDa rescued TTX-induced homeostatic synaptic plasticity in these experiments, while in the presence of conditioned medium with larger peptides no synaptic scaling was observed in *APP^−/−^* preparations (Fig. 5*D*,*E*). We conclude from these results that a small (i.e., <10 kDa) secreted molecular factor originating from *APP^+/+^* tissue is sufficient to rescue the ability of granule cells in the *APP*^−/−^ cultures to express TTX-induced homeostatic synaptic plasticity. The results of these experiments also indicate that the presence of APP in the target region is not required (i.e., APP per se does not act as a receptor for signaling pathways relevant for TTX-induced excitatory synaptic scaling).

### APPsα does not rescue homeostatic synaptic plasticity in APP-deficient preparations

Previous work revealed that APPsα rescues several phenotypes observed in *APP*^−/−^ mice, including alterations in LTP (e.g., Ring et al., 2007; Hick et al., 2015; Fol et al., 2016; Richter et al., 2018). We therefore tested whether APPsα rescues the ability of *APP*^−/−^ granule cells to express homeostatic synaptic plasticity in our experiments.

**Figure 6.**
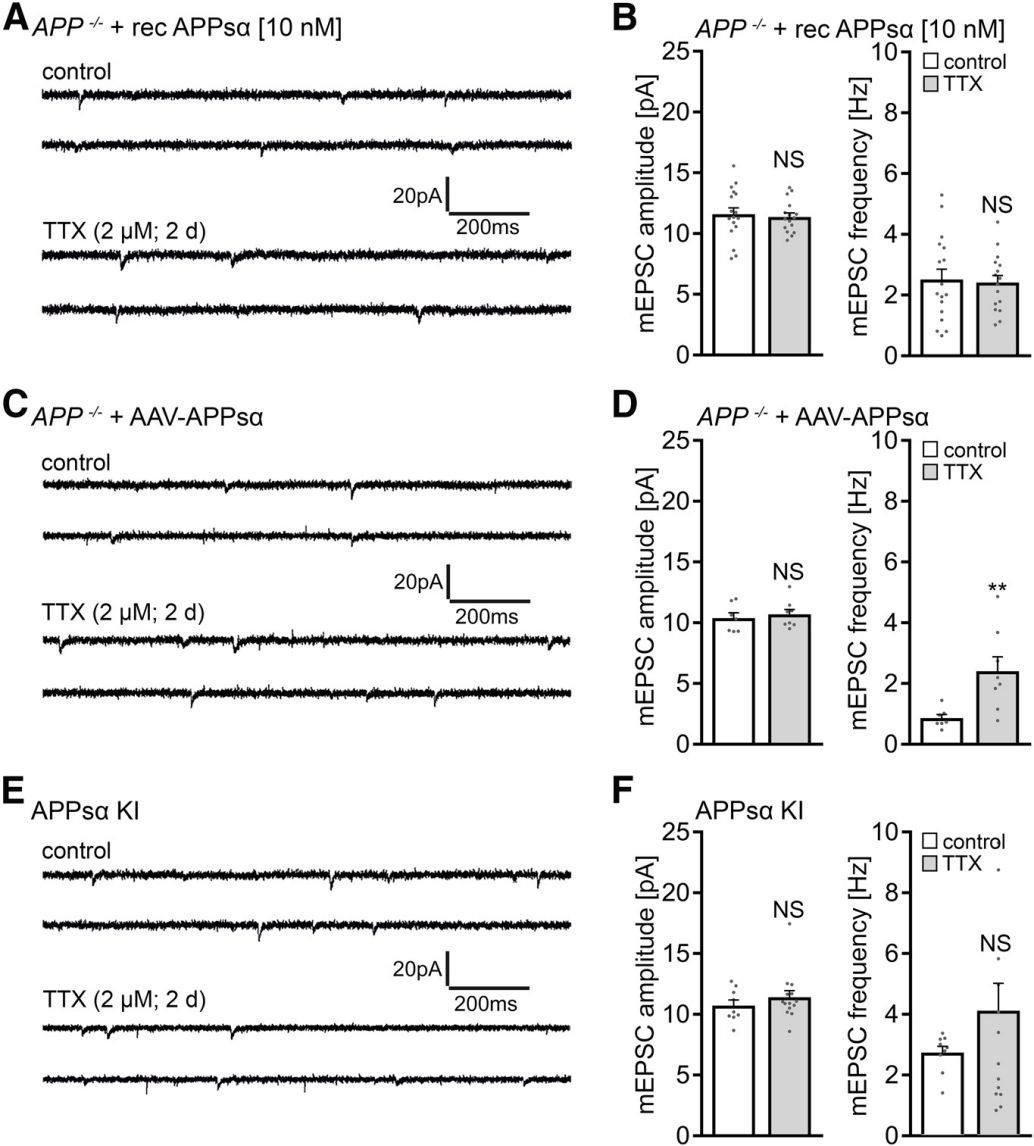
APPsα does not rescue the ability of APP-deficient dentate granule cells to express homeostatic synaptic plasticity. ***A***, ***B***, Sample traces and group data of AMPAR-mediated mEPSCs recorded from granule cells in vehicle-treated (control) and TTX-treated (2 μm, 2 d) *APP^−/−^* tissue cultures in the presence of recombinant APPsα (10 nm; control, *n* = 17 cells from 5 cultures; TTX, *n* = 16 cells from 5 cultures; Mann–Whitney test). ***C***, ***D***, Sample traces and group data of AMPAR-mediated mEPSCs from granule cells in *APP^−/−^* tissue cultures transduced with AAV vectors expressing APPsα (control, *n* = 7 cells; TTX, *n* = 8 cells; from 2 cultures per group; Mann–Whitney test). ***E***, ***F***, Sample traces and group data of AMPAR-mediated mEPSCs from granule cells in tissue cultures prepared from APPsα knock-in (*APPsα KI*) mice (control, *n* = 9 cells from 3 cultures; TTX, *n* = 14 cells from 4 cultures; Mann–Whitney test). For mEPSC frequency, one data point is outside the axis limits in the TTX group. Data are mean ± SEM. NS, Not significant. **p < 0.01.

First, *APP*^−/−^ cultures were treated with APPsα at a concentration that rescues LTP in acute hippocampal slices (10 nm) (Hick et al., 2015), and TTX-induced synaptic scaling was probed. In these experiments, no homeostatic synaptic adjustment was observed (Fig. 6*A*,*B*), thus confirming once more our major finding (i.e., alterations in homeostatic synaptic plasticity of *APP*^−/−^ dentate granule cells).

We next resorted to AAV transduction of secreted APPsα (AAV-Syn-T2A-Venus-APPsα) using the same protocol as described for flAPP (compare Fig. 1*H*,*I*). The expression of this viral construct was previously used to successfully rescue LTP defects in *APP*^−/−^ mice (Richter et al., 2018). Again, in our experimental setting, no compensatory increase in mean mEPSC amplitude was observed after TTX treatment compared with age- and time-matched vehicle-only-treated APPsα-transfected *APP*^−/−^ tissue cultures (Fig. 6*C*,*D*). An increase in mEPSC frequencies back to baseline was detected in the TTX group in these experiments, indicating complex effects of viral transduction on mEPSC frequencies in the dentate gyrus (Fig. 6*D*) (see also Lazarevic et al., 2017; Rice et al., 2019). However, viral transduction per se does not rescue the ability of *APP*^−/−^ granule cells to express homeostatic synaptic plasticity (compare Fig. 1*I*).

Finally, tissue cultures from *APPsα-KI* mice were prepared (Ring et al., 2007), which express APPsα constitutively while lacking transmembrane APP and Aβ; this represents another approach for rescuing LTP (Ring et al., 2007). Because in these experiments we also did not observe homeostatic plasticity (Fig. 6*E*,*F*), we conclude that APPsα does not rescue TTX-induced homeostatic synaptic plasticity of dentate granule cells in *APP^−/−^* tissue cultures, at least not by using the above-described experimental approaches that all rescue LTP.

### Scavenging endogenous APPsα with a specific antibody does not block homeostatic synaptic plasticity

**Figure 7.**
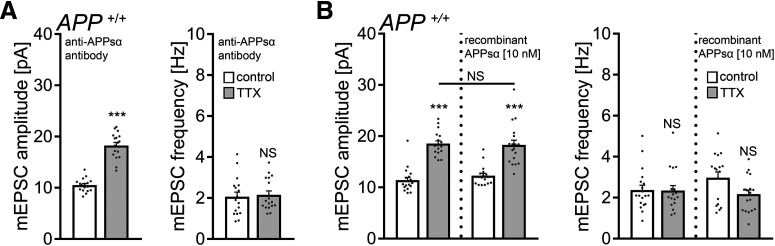
APPsα has no apparent effect in TTX-induced homeostatic plasticity of WT dentate granule cells. ***A***, Group data of AMPAR-mediated mEPSCs recorded from granule cells in vehicle-treated (control) and TTX-treated (2 μm, 2 d) *APP^+/+^* tissue cultures in the presence of a specific anti-APPsα antibody (JRD32; 1.3 μg/ml; *n* = 17 cells from 6 cultures per group; Mann–Whitney test). ***B***, Recombinant APPsα (10 nm) does not cause aberrant “over”-scaling of *APP^+/+^* dentate granule cells (control, *n* = 19 cells from 7 cultures; TTX, *n* = 18 cells from 6 cultures; APPsα, *n* = 17 cells from 6 cultures; TTX + APPsα, *n* = 18 cells from 6 cultures). Data are mean ± SEM. NS, Not significant. ***p < 0.001 (Kruskal–Wallis test followed by Dunn’s *post hoc* test).

We next tested for the effects of endogenous APPsα by treating WT tissue cultures with TTX (2 μm; 2 d) in the presence of a specific antibody that binds APPs (i.e., the N-terminal APP-E1 domain; JRD32; 1.3 μg/ml) (Hick et al., 2015). As shown in Figure 7*A*, a compensatory increase in mEPSC amplitudes was observed in these experiments, thus providing additional evidence that APPsα is not involved in mediating homeostatic synaptic plasticity.

Because treatment with recombinant APPsα (10 nm) also did not cause aberrant “over”-scaling in *APP^+/+^* dentate granule cells (Fig. 7*B*), together with the experiments conducted in *APP*^−/−^ cultures (compare Figs. 5 and 6), we are confident to conclude that APPsα is not a major regulator of homeostatic synaptic plasticity (this study), whereas it does promote LTP (e.g., Ring et al., 2007; Hick et al., 2015; Fol et al., 2016; Richter et al., 2018).

### Aβ rescues homeostatic synaptic plasticity in APP-deficient preparations

**Figure 8.**
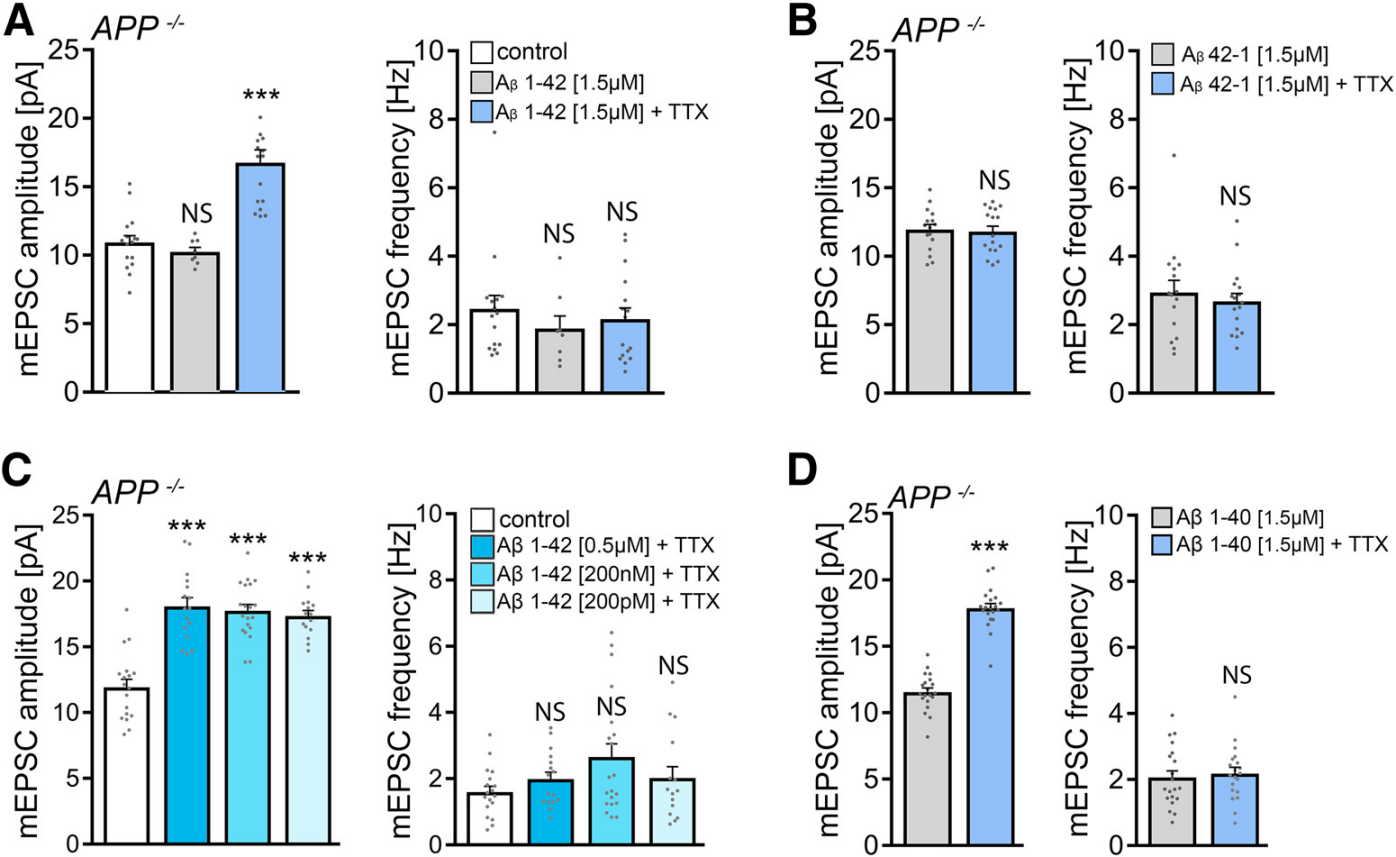
Amyloid-β_1-42_ rescues the ability of APP-deficient dentate granule cells to express homeostatic synaptic plasticity. ***A***, ***B***, Group data of AMPAR-mediated mEPSCs recorded from granule cells in vehicle-treated (control) and TTX-treated (2 μm, 2 d) *APP*^−/−^ tissue cultures in the presence of amyloid-β_1-42_ (Aβ_1-42_; 1.5 μΜ, ***A***) or Aβ_42-1_ (1.5 μΜ, ***B***). ***A***, Control, *n* = 16 cells from 4 cultures; Aβ_1-42_, *n* = 8 cells from 3 cultures; Aβ_1-42_ + TTX, *n* = 16 cells from 5 cultures; Kruskal–Wallis test followed by Dunn’s *post hoc* test; for mEPSC amplitudes, one data point is outside the axis limits in the Aβ_1-42_ + TTX group. ***B***, Aβ_42-1_, *n* = 16 cells from 4 cultures; Aβ_42-1_ + TTX, *n* = 17 cells from 5 cultures; Mann–Whitney test. ***C***, Group data of AMPAR-mediated mEPSCs recorded from granule cells in vehicle-treated (control) and TTX-treated (2 μm, 2 d) *APP*^−/−^ tissue cultures in the presence of 0.5 μm, 200 nm, and 200 pm of Aβ_1-42_ (control, *n* = 18 cells from 5 cultures; 0.5 μm Aβ_1-42_ + ΤΤΧ, *n* = 16 cells from 4 cultures; 200 nm Aβ_1-42_ + TTX, *n* = 20 cells from 6 cultures; 200 pm Aβ_1-42 +_ TTX, *n* = 15 cells from 4 cultures; Kruskal–Wallis test followed by Dunn’s *post hoc* test). ***D***, Group data of AMPAR-mediated mEPSCs recorded from *APP*^−/−^ granule cells in the presence of 1.5 μΜ Aβ_1-40_ (*n* = 20 cells from 6 cultures in each group; Mann–Whitney test). Data are mean ± SEM. NS, Not significant. ***p < 0.001.

We then considered the amyloidogenic processing pathway and the potential for Aβ involvement in mediating homeostatic synaptic plasticity. Another set of *APP*^−/−^ tissue cultures was treated for 2 d with 2 μm TTX and with a synthetic Aβ protein fragment 1-42 (1.5 μm) (Novotny et al., 2016). Notably, a full-sized homeostatic synaptic scaling response was observed in these experiments (Fig. 8*A*): in the presence of Aβ_1-42_, mEPSC amplitudes increased from 10.9 ± 0.5 pA to 16.8 ± 0.9 pA after 2 d TTX treatment (*p* < 0.001; Kruskal–Wallis test followed by Dunn’s *post hoc* test). Because similar experiments with the synthetic inverse Aβ protein fragment 42-1 (1.5 μm) did not show any significant changes in mEPSC properties (Fig. 8*B*), we conclude that the application of exogenous Aβ_1-42_ rescues TTX-induced homeostatic synaptic plasticity in *APP*^−/−^ preparations.

We also explored possible dose-dependent effects of Αβ_1-42_ at concentrations of 0.5 μm, 200 nm, and 200 pm (compare Puzzo et al., 2008), and we found comparable TTX-induced synaptic strengthening at all concentrations tested (Fig. 8*A*). Moreover, increased mEPSC amplitudes were also observed in the presence of Aβ_1-40_ (1.5 μΜ; Fig. 8*D*). We conclude from these results that exogenous Aβ rescues the ability of *APP^−/−^* dentate granule cells to express homeostatic synaptic plasticity.

### Pharmacological inhibition of β-secretases in WT tissue cultures blocks homeostatic synaptic plasticity

**Figure 9.**
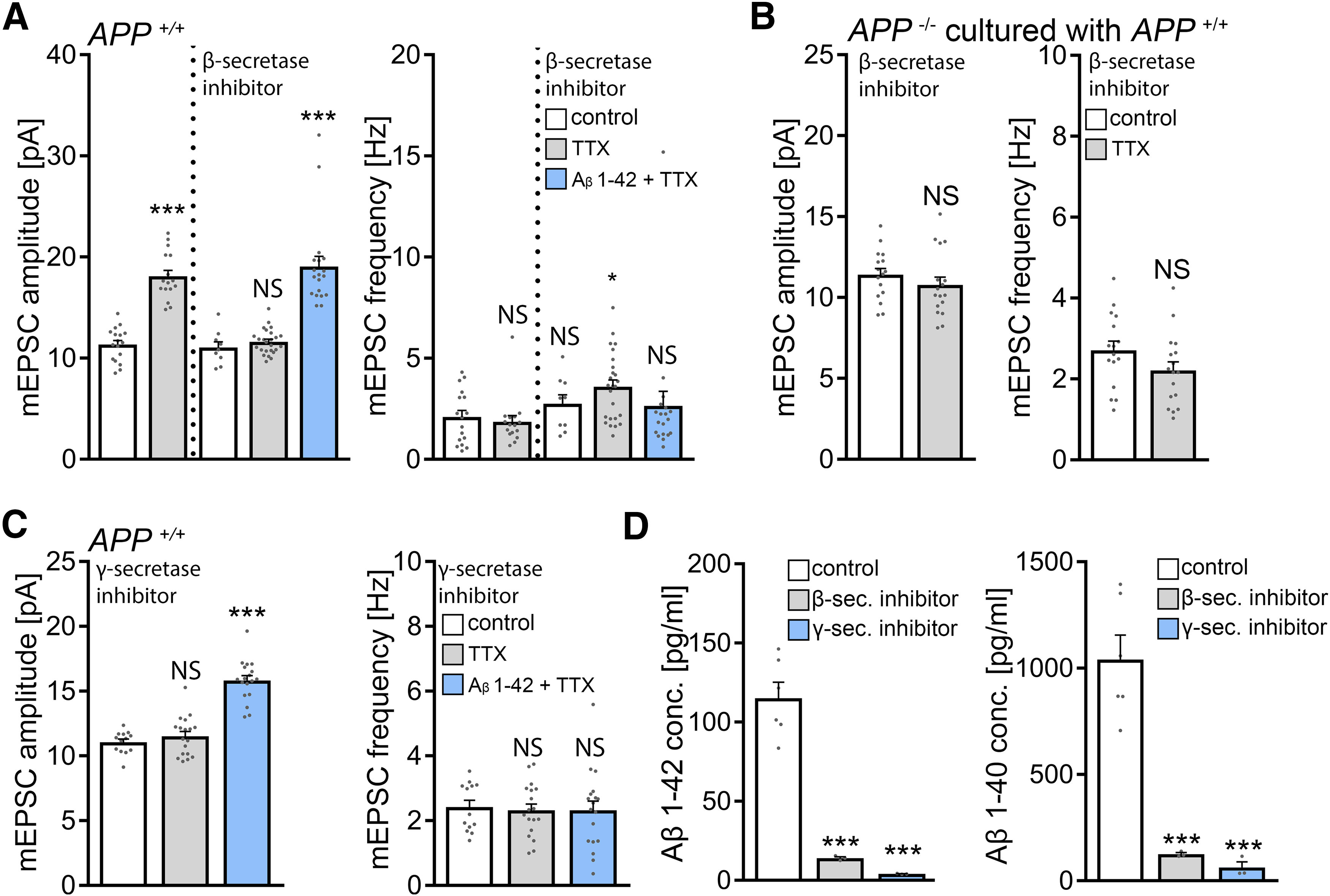
Pharmacological inhibition of β- or γ-secretases blocks homeostatic synaptic plasticity. ***A***, Group data of AMPAR-mediated mEPSCs recorded from granule cells in vehicle-treated (control) and TTX-treated (2 μm, 2 d) WT tissue cultures. BACE inhibitor C3 (20 μm) prevents TTX-induced synaptic scaling, an effect that is reversed in the presence of Aβ_1-42_ (1.5 μm). Untreated: control, *n* = 17 cells from 5 cultures; TTX, *n* = 16 cells from 4 cultures. β-secretase inhibitor: control, *n* = 9 cells from 3 cultures; TTX, *n* = 25 cells from 7 cultures; Aβ_1-42_ + TTX, *n* = 19 cells from 5 cultures; Kruskal–Wallis test followed by Dunn’s *post hoc* test. ***B***, Group data of AMPAR-mediated mEPSCs recorded from granule cells in vehicle-treated (control) and TTX-treated (2 μm, 2 d) *APP*^−/−^ preparations co-cultured with *APP^+/+^* tissue (compare Fig. 5). BACE inhibitor C3 (20 μm) prevents TTX-induced synaptic scaling (control, *n* = 16 cells from 5 cultures; TTX, *n* = 17 cells from 5 cultures; Mann–Whitney test). ***C***, Pharmacological inhibition of γ-secretases with Begacestat (1 μm) prevents TTX-induced synaptic scaling of dentate granule cells in WT tissue cultures, an effect that is reversed in the presence of Aβ_1-42_ (1.5 μm). Control, *n* = 13 cells from 4 cultures; TTX, *n* = 18 cells from 6 cultures; Aβ_1-42_ + TTX, *n* = 18 cells from 6 cultures; Kruskal–Wallis test followed by Dunn’s *post hoc* test. ***D***, Concentrations of Aβ_1-42_ and Aβ_1-40_ in culturing medium collected from vehicle-treated (control), BACE inhibitor C3-treated (β-sec. inhibitor), and Begacestat-treated (γ-sec. inhibitor) *APP*^+/+^ tissue cultures (*n* = 3-6 wells per group; one-way ANOVA). Data are mean ± SEM. NS, Not significant. *p < 0.05. ***p < 0.001.

Next, we sought to test for the role of endogenous Aβ on homeostatic synaptic plasticity. Because Aβ is produced by sequential cleavage of APP by β- and γ-secretases (Chow et al., 2010; Zheng and Koo, 2011), we hypothesized that pharmacological inhibition of β-secretases (i.e., blocking the first step of the amyloidogenic processing pathway) should impede the ability of WT dentate granule cells to express TTX-induced synaptic scaling. Accordingly, WT cultures were treated with TTX (2 μm; 2 d) and with BACE inhibitor C3 (20 μm; 2 d) (Stachel et al., 2004). Pharmacological inhibition of β-secretases had no apparent effect on baseline mEPSC recordings, and a TTX-induced increase in mEPSC amplitudes was not observed. Exposure to Aβ_1-42_ (1.5 μm) together with BACE inhibitor C3 restored the ability of WT dentate granule cells to express homeostatic synaptic scaling (Fig. 9*A*). Furthermore, in the presence of BACE inhibitor C3, TTX-induced synaptic scaling was not observed in *APP*^−/−^ preparations co-cultured with 5 *APP^+/+^* cultures on the same membrane insert (Fig. 9*B*; compare Fig. 5). These results suggest that APP/Aβ is part of an endogenous signaling pathway that mediates homeostatic synaptic plasticity.

### Pharmacological inhibition of γ-secretases in WT tissue cultures blocks homeostatic synaptic plasticity

BACE not only cleaves APP but also targets several other substrates in the nervous system (Barao et al., 2016). Although Aβ_1-42_ rescued homeostatic synaptic plasticity in BACE inhibitor C3-treated WT cultures, we decided to err on the side of caution. Accordingly, another series of experiments was conducted using the γ-secretase inhibitor Begacestat (GSI-953; 1 μm) (Martone et al., 2009) to block the second enzymatic step of the amyloidogenic processing pathway. As shown in Figure 9*C*, a compensatory increase in mEPSC amplitudes was not observed in the presence of Begacestat after TTX treatment. Again, Aβ_1-42_ (1.5 μm) rescued the ability of WT dentate granule cells to express homeostatic synaptic plasticity in the presence of the γ-secretase inhibitor (Fig. 9*C*). Consistent with these findings, Aβ sandwich-ELISA confirmed that endogenous Aβ_1-40_ and Aβ_1-42_ are significantly reduced by pharmacological inhibition of β- or γ-secretases in our experimental setting (Fig. 9*D*).

### Scavenging endogenous Aβ with a specific antibody blocks homeostatic synaptic plasticity in WT tissue cultures

**Figure 10.**
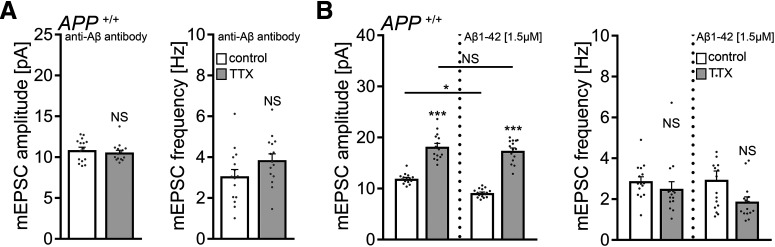
Endogenous Aβ is required for the expression of homeostatic synaptic plasticity. ***A***, Group data of AMPAR-mediated mEPSCs recorded from granule cells in vehicle-treated (control) and TTX-treated (2 μm, 2 d) *APP^+/+^* tissue cultures in the presence of a specific anti-Aβ antibody (BNT-77; 1.3 μg/ml; *n* = 15 cells from 5 cultures in each group; Mann–Whitney test). ***B***, Group data of AMPAR-mediated mEPSCs recorded from granule cells in vehicle-treated (control) and TTX-treated (2 μm, 2 d) *APP^+/+^* tissue cultures in the presence of Aβ_1-42_ (1.5 μm). Αβ_1-42_ does not cause aberrant “over”-scaling (compare with Fig. 7; untreated group: *n* = 15 cells from 5 cultures in each group; Aβ_1-42_ [1.5 μm] treated group: control, *n* = 16 cells from 5 cultures; TTX, *n* = 15 cells from 5 cultures; Kruskal–Wallis test followed by Dunn’s *post hoc* test). Data are mean ± SEM. NS, Not significant. *p < 0.5. ***p < 0.001.

Finally, we conducted experiments in WT cultures using an antibody that binds endogenous Aβ (anti-Aβ_11-28_; BNT77; 1.3 μg/ml) (Hashimoto et al., 2010). No significant differences in mEPSC properties between vehicle-only and TTX-treated WT dentate granule cells were observed in these experiments (Fig. 10*A*). Similarly, in the experimental setting in which *APP*^−/−^ tissue was co-cultured with *APP^+/+^* cultures, again, no TTX-induced synaptic scaling was observed in the *APP*^−/−^ cultures in the presence of the anti-Aβ antibody (control, 10.5 ± 0.3 pA; TTX, 9.9 ± 0.2 pA; Mann–Whitney test; *p* = 0.09; *n* = 16 cells from 4 cultures in each group; compare Fig. 5). We also tested for the effects of exogenous Aβ_1-42_ (1.5 μm) on TTX-induced homeostatic synaptic plasticity in WT preparations and found no signs for aberrant “over”-scaling (Fig. 10*B*) (compare Gilbert et al., 2016). Consistent with earlier reports, a reduction in mEPSC amplitudes was observed in Aβ_1-42_ treated controls (Fig. 10*B*) (Chang et al., 2006; Gilbert et al., 2016).

Together, we conclude that APP is a key regulator of homeostatic synaptic plasticity and that Aβ-dependent signaling pathways account for the ability of cultured dentate granule cells to express TTX-induced homeostatic plasticity of excitatory synapses.

### Pharmacological inhibition of NMDARs rescues homeostatic synaptic plasticity in APP-deficient preparations

What are the downstream signaling pathways of APP/Aβ-mediated homeostatic synaptic plasticity? Previous work revealed that Aβ affects Hebbian plasticity via modulation of NMDARs (Chen et al., 2002; Snyder et al., 2005; Zhang et al., 2009). Considering the role of NMDARs in homeostatic synaptic plasticity (i.e., NMDAR inhibition triggers synaptic up-scaling) (Sutton et al., 2006), we hypothesized that Aβ may trigger AMPAR-mediated homeostatic synaptic plasticity by modulating NMDARs.

**Figure 11.**
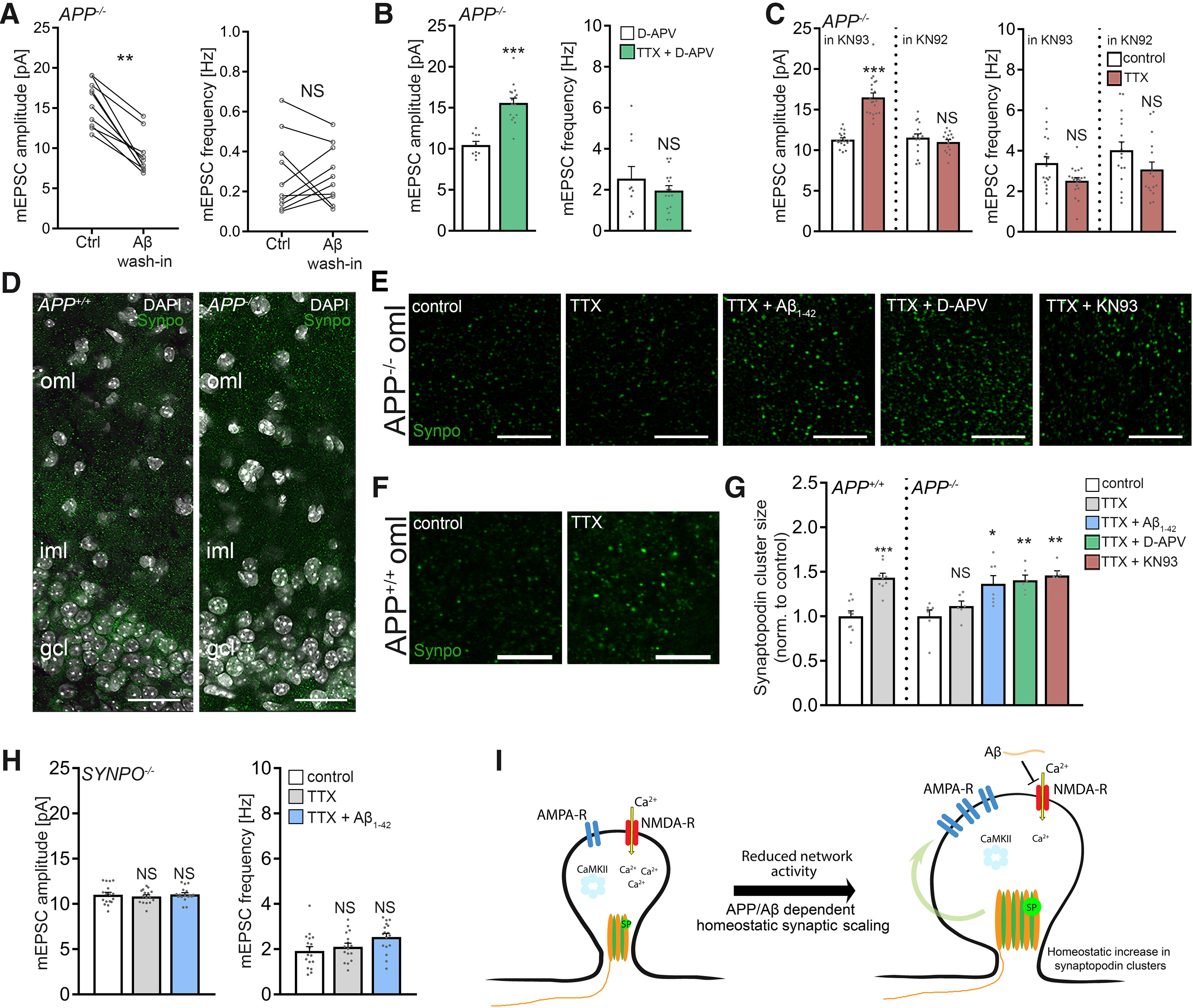
Role of Ca^2+^ signaling and synaptopodin in APP/amyloid-β-mediated homeostatic synaptic scaling. ***A***, Group data of NMDAR-mediated mEPSCs recorded from *APP^−/−^* dentate granule cells before and after washing Αβ_1-42_ (1.5 μm) into the recording chamber (*n* = 10 cells from 5 tissue cultures; Wilcoxon test). ***B***, Group data of AMPAR-mediated mEPSCs recorded from granule cells in D-APV (50 μm; 2 d)-treated and D-APV (50 μm) + TTX-treated (2 μm, 2 d) *APP*^−/−^ tissue cultures (D-APV, *n* = 10 cells from 3 cultures; TTX + D-APV, *n* = 16 cells from 4 cultures; Mann–Whitney test). ***C***, Group data of AMPAR-mediated mEPSCs from granule cells in *APP*^−/−^ cultures recorded in the presence of the CaMKII inhibitor KN93 or the inactive analog KN92 (KN93: control, *n* = 18 cells from 5 cultures; TTX, *n* = 20 cells from 5 cultures; KN92: control, *n* = 17 cells from 5 cultures; TTX = *n* = 16 cells from 5 cultures; Mann–Whitney test). ***D***, Example of *APP^+/+^* and *APP*^−/−^ tissue cultures immunostained for synaptopodin (Synpo, green). Cluster sizes were assessed in the outer molecular layer (oml) of the dentate gyrus. iml, Inner molecular layer; gcl, granule cell layer. DAPI, nuclear stain. Scale bar, 50 μm. ***E***, ***F***, Examples of analyzed visual fields (six fields per culture and condition) at higher magnification. All treatments 2 d. TTX, 2 μm; Aβ_1-42_, 1.5 μm; D-APV, 50 μm, KN93, 5 μm. Scale bar, 5 µm. ***G***, Group data of synaptopodin cluster sizes in the respective groups. Values normalized to controls (*APP^+/+^* *n* = 9 cultures per group; *APP^−/−^* control, *n* = 7 cultures; TTX, *n* = 6 cultures; TTX + Aβ_1-42_, *n* = 7 cultures; TTX + D-APV, *n* = 6 cultures; TTX + KN93, *n* = 6 cultures; *APP^+/+^* Mann–Whitney test; *APP^−/−^*, Kruskal–Wallis test followed by Dunn’s *post hoc* test). ***H***, Group data of AMPAR-mediated mEPSCs recorded from granule cells in synaptopodin-deficient (*SYNPO*^−/−^) tissue cultures. Aβ_1-42_ (1.5 μm, 2 d) does not rescue TTX-induced homeostatic synaptic plasticity (untreated, *n* = 17 cells from 6 cultures; TTX, *n* = 16 cells from 5 cultures; TTX + Aβ_1-42_, *n* = 17 cells from 5 cultures; Kruskal–Wallis test followed by Dunn’s *post hoc* test). ***I***, Model of APP/Αβ-mediated homeostatic synaptic scaling. APP/Aβ is part of a Ca^2+^-dependent negative feedback mechanism that regulates synaptopodin cluster properties and synaptic accumulation of AMPAR in an NMDAR- and CaMKII-dependent manner. Plastic changes in synaptopodin cluster sizes and spine apparatuses were previously linked to synaptic plasticity (compare Vlachos et al., 2009; 2012; Lenz et al., 2021). Data are mean ± SEM. NS, Not significant. *p < 0.05. **p < 0.01. ***p < 0.001.

To test for the effects of Αβ on NMDARs in our experiments, NMDAR-mediated mEPSCs were recorded from *APP^−/−^* dentate granule cells at a holding potential +40 mV, and Αβ_1-42_ (1.5 μm) was washed into the recording chamber. A significant reduction in NMDAR-mediated mEPSC amplitudes was observed in these experiments (Fig. 11*A*). Based on these results, we speculated that pharmacological inhibition of NMDARs could rescue the ability of *APP^−/−^* dentate granule cells to express TTX-induced synaptic scaling. Indeed, in the presence of the NMDAR antagonist D-APV (50 μm), a significant increase in AMPAR-mediated mEPSC amplitudes was noted in TTX-treated *APP^−/−^* preparations, consistent with a compensatory (i.e., homeostatic) synaptic response (Fig. 11*B*). In a separate experiment, baseline recordings of NMDAR-mediated mEPSCs showed no significant differences between untreated *APP^−/−^* and *APP^+/+^* granule cells (*APP^+/+^*, 17.6 ± 0.7 pA; *APP^−/−^*, 17.0 ± 0.6 pA; *n* = 9 and *n* = 10 cells, respectively; *p* = 0.66; Mann–Whitney test), confirming once more that basic functional differences between the two genotypes do not trivially explain our major findings (compare Figs. 3 and 4).

### Pharmacological inhibition of CaMKII rescues homeostatic synaptic plasticity in APP-deficient preparations

Aβ has been linked to inactivation of CaMKII (Zhao et al., 2004; Townsend et al., 2007; Gu et al., 2009; but see Opazo et al., 2018). Hence, we reasoned that, in the absence of APP/Aβ, pharmacological inhibition of CaMKII might also rescue TTX-induced synaptic scaling. Indeed, increased mEPSC amplitudes were observed in *APP*^−/−^ cultures after TTX treatment in the presence of KN-93 (5 μm), but not with the inactive analog KN-92 (5 μm; Fig. 11*C*). Hence, NMDAR-mediated CaMKII-dependent downstream signaling pathways seem to be involved in APP/Aβ-mediated homeostatic synaptic plasticity.

### Role of synaptopodin in APP/Aβ-mediated homeostatic synaptic plasticity

In previous work, we demonstrated that the actin-binding molecule synaptopodin, which is a marker and essential component of the Ca^2+^-storing spine apparatus organelle (Deller et al., 2003), is required for the expression of homeostatic synaptic plasticity (Vlachos et al., 2013). In this context, a Ca^2+^-dependent compensatory increase in synaptopodin clusters was observed, consistent with a negative feedback mechanism that mediates homeostatic synaptic plasticity (Vlachos et al., 2013). Notably, an association between synaptopodin and peptides corresponding to CaMKIIα and CaMKIIβ has been recently reported (Konietzny et al., 2019).

To test whether activity-dependent changes of synaptopodin are part of a negative feedback mechanism that requires APP/Aβ-dependent signaling, *APP*^−/−^ cultures were once again treated with TTX (2 μm; 2 d). Immunostained synaptopodin clusters were assessed in the outer molecular layer of the dentate gyrus (Fig. 11*D–F*), and the previously reported compensatory increase in synaptopodin cluster sizes was not observed in TTX-treated *APP*^−/−^ cultures (Fig. 11*G*) (compare Vlachos et al., 2013). We then tested whether, in the presence of Aβ_1-42_ (1.5 μm), TTX-induced changes in synaptopodin clusters are triggered. Indeed, the same protocol that rescues homeostatic synaptic plasticity in *APP*^−/−^ preparations also induces synaptopodin changes similar to that observed in WT cultures (Fig. 11*G*).

Does pharmacological inhibition of NMDARs and CaMKII rescue homeostatic changes in synaptopodin clusters of *APP*^−/−^ cultures? As shown in Figure 11*G*, in the presence of D-APV (50 μm) or KN93 (5 μm), a significant increase in synaptopodin clusters was observed in response to TTX (2 μm, 2 d). These results indicate that APP/Aβ is part of a Ca^2+^-dependent negative feedback mechanism that regulates synaptopodin cluster properties in an NMDAR- and CaMKII-dependent manner.

Finally, based on the observation that Aβ_1-42_ rescues TTX-induced homeostatic synaptic plasticity, we tested for the effects of Aβ_1-42_ in synaptopodin-deficient dentate granule cells, which do not form spine apparatus organelles and show defects in homeostatic synaptic plasticity (Vlachos et al., 2013). Whereas the previously reported deficit in TTX-induced homeostatic synaptic plasticity was reproduced in these experiments, Aβ_1-42_ did not rescue homeostatic synaptic plasticity in *SYNPO*^−/−^ dentate granule cells (Fig. 11*H*). Together, we propose that synaptopodin is one of the downstream molecular targets required for APP/Aβ-mediated homeostatic synaptic plasticity (Fig. 11*I*).

## Discussion

We regard the significant finding of this study to be the discovery of a previously unknown APP/Aβ phenotype that becomes salient after network activity is altered (i.e., blocked by TTX for a prolonged period of time). Under these conditions, *APP*^−/−^ dentate granule cells do not scale their excitatory synapses in a homeostatic manner. Surprisingly, this plasticity phenotype does not depend on APPsα, which is generated from APP via nonamyloidogenic processing and which has been linked to Hebbian plasticity, but on Aβ, which is generated from APP via processing along the amyloidogenic pathway. Consistent with this observation, pharmacological inhibition of β- and γ-secretases as well as scavenging endogenous Aβ with antibodies blocked TTX-induced synaptic scaling in WT tissue cultures. Aβ-dependent synaptic scaling required modulation of downstream Ca^2+^-dependent signaling pathways, including NMDARs and CaMKII, as well as synaptopodin, a molecule essential for the formation of the Ca^2+^-storing spine apparatus. Together, these results reveal a direct involvement of amyloidogenic APP processing and Aβ in homeostatic synaptic plasticity. This involvement raises the intriguing possibility that changes in the balance of APP processing along the two major APP-processing pathways could also lead to changes in the balance of Hebbian and homeostatic plasticity in the brain (compare Galanis and Vlachos, 2020).

Studies using mouse mutants lacking APP or APP-gene family members have established a firm link between APP and the ability of neurons to express synaptic plasticity (Müller et al., 2017). Specifically, the ability of neurons to express LTP of excitatory neurotransmission is impaired in aged *APP*^−/−^ mice (e.g., Dawson et al., 1999; Seabrook et al., 1999; Ring et al., 2007; Tyan et al., 2012). APPsα could rescue these deficits (e.g., Ring et al., 2007; Hick et al., 2015; Fol et al., 2016; Richter et al., 2018) using recombinant, viral, and genetic strategies, suggesting a “plasticity-promoting” role of APP via the non-amyloidogenic processing pathway (Mockett et al., 2017).

Based on these observations, we assumed that another major form of synaptic plasticity (i.e., homeostatic synaptic plasticity) (Turrigiano et al., 1998) could also depend on APP or one of its diverse processing products. Indeed, *APP*^−/−^ neurons failed to scale their synapses after TTX treatment, implicating APP in homeostatic plasticity, but APPsα could not rescue this APP-dependent plasticity phenotype. Similarly, scavenging endogenous APPsα or treatment with recombinant APPsα did not affect TTX-induced homeostatic synaptic plasticity in WT dentate granule cells. This phenomenon cannot be trivially explained by an inability of dentate granule cells to respond to APPsα because the exogenous application of APPsα robustly enhances LTP in these neurons (Taylor et al., 2008). Together, these observations suggest that the plasticity-promoting effect of APPsα (Mockett et al., 2017) should be narrowed down and specified: APP processing via the non-amyloidogenic pathway promotes the ability of neurons to express LTP, but not necessarily all forms of synaptic plasticity because our data demonstrate that APPsα is not a key regulator of homeostatic synaptic plasticity.

The inability to rescue homeostatic plasticity via APPsα (compare Fig. 6) raised the intriguing possibility that APP processing via the “amyloidogenic pathway” could serve homeostatic synaptic plasticity. In the presence of Aβ (i.e., Aβ_1−40_ and Aβ_1−42_), the homeostatic synaptic response to TTX was fully restored in *APP^−/−^* preparations. Moreover, we report that pharmacological inhibition of β-secretases blocks TTX-induced homeostatic synaptic plasticity in WT cultures. Because pharmacological inhibition of γ-secretase had a similar effect, and Aβ rescued homeostatic synaptic plasticity in the presence of β- or γ-secretase inhibitors, other APP cleavage products [e.g., APPsβ (or additional factors arising from β- or γ-secretase processing)] are unlikely to account for our significant findings. In a previous study, however, pharmacological inhibition of γ-secretases did not block TTX-induced homeostatic synaptic plasticity (Pratt et al., 2011). These earlier experiments were conducted in primary hippocampal neurons rather than in organotypic tissue cultures, and L685458 (5 μm) was used instead of Begacestat (1 μm) in our study. These differences may explain the inconsistent results. Notably, we confirmed that pharmacological inhibition of γ-secretase with Begacestat reduced endogenous Aβ levels in our experimental setting, and exogenous Aβ_1-42_ rescued homeostatic synaptic plasticity in Begacestat-treated tissue cultures.

To provide further evidence for the role of Aβ in neuronal physiology, we used antibodies against Aβ and scavenged endogenously produced Aβ from WT tissue cultures. This approach abolished homeostatic synaptic scaling in the WT cultures and prevented synaptic scaling in *APP*^−/−^ preparations cultured together with *APP^+/+^* tissue, whereas an antibody against APPsα did not affect TTX-induced synaptic scaling. Together, we propose a model in which APP processing via the non-amyloidogenic pathway and the amyloidogenic pathway influences the ability of a neuron to express distinct forms of synaptic plasticity: processing along the non-amyloidogenic pathway may promote Hebbian plasticity (e.g., LTP), whereas processing along the amyloidogenic pathway, or increased availability of Aβ (Gilbert et al., 2016), promotes homeostatic synaptic plasticity.

Interestingly, no major alterations in dendritic arborization, dendritic spines, PSDs, and spine apparatus organelles were observed in the dentate gyrus of *APP^−/−^* preparations (Fig. 4). Previous work demonstrated structural defects in *APP^−/−^* CA1 pyramidal neurons (both *in vitro* and *in vivo*) (Wei et al., 2010; Tyan et al., 2012; Weyer et al., 2014). While additional work is required to determine the reasons for such region-specific difference (e.g., differential expression of APP) (Del Turco et al., 2016), we can state with confidence that major structural and functional alterations do not readily explain the major findings of the present study, also considering the experiments performed with WT tissue. Indeed, no differences in sEPSCs and sIPSCs, basic intrinsic properties, and synaptic NMDAR currents were observed in cultured dentate granule cells under baseline conditions between the genotypes.

What are the downstream pathways activated by Aβ in the context of homeostatic plasticity? Consistent with previous work on Hebbian plasticity (Zhao et al., 2004; Snyder et al., 2005; Gu et al., 2009; Zhang et al., 2009; Sinnen et al., 2016), we could link the homeostatic plasticity effects of Aβ to NMDARs and CaMKII. Considering the opposing roles of NMDARs and CaMKII in Hebbian and homeostatic synaptic plasticity, we speculated that Aβ signaling could be part of a Ca^2+^-dependent negative feedback mechanism that mediates homeostasis. Indeed, pharmacological inhibition of NMDARs and CaMKII rescued homeostatic synaptic plasticity in *APP*^−/−^ preparations, suggesting that Aβ acts on intracellular Ca^2+^ sensors or effectors, which responded to the TTX-induced reduction in intracellular Ca^2+^ in our experiments. Consistent with this suggestion, the previously reported Ca^2+^-dependent compensatory adjustment of synaptopodin was not observed in *APP*^−/−^ preparations (compare Vlachos et al., 2013), and interventions that rescued TTX-induced synaptic scaling in *APP*^−/−^ dentate granule cells also triggered compensatory changes in synaptopodin. Moreover, Aβ was not able to rescue homeostatic synaptic plasticity in *SYNPO*^−/−^ preparations, demonstrating that synaptopodin is an essential downstream target through which APP/Aβ asserts its effects on homeostatic synaptic plasticity.

In previous work, we have shown that the plasticity-related protein synaptopodin controls the ability of neurons to express both Hebbian and homeostatic synaptic plasticity (Deller et al., 2003; Jedlicka et al., 2009; Vlachos et al., 2009, 2013). Animals lacking this protein do not form spine apparatus organelles and exhibit deficits in Hebbian and homeostatic synaptic plasticity (Deller et al., 2003; Jedlicka et al., 2009; Vlachos et al., 2013) because the Ca^2+^-dependent accumulation of AMPARs at excitatory postsynapses is impaired (e.g., Vlachos et al., 2009, 2013; Maggio and Vlachos, 2018). The results of the present study call for a systematic assessment of the activity-dependent molecular pathways through which APP processing via the “amyloidogenic/homeostatic” and “non-amyloidogenic/Hebbian plasticity promoting” pathways affects synaptopodin-mediated synaptic plasticity. It may be important to mention in this context that changes in synaptopodin expression have been reported in brain tissue from AD subjects (Reddy et al., 2005), and synaptopodin has been recently linked to autophagy of phospho-MAPT/Tau (Ji et al., 2019). Moreover, a reduction in synaptopodin expression seems to ameliorate symptoms in a transgenic mouse model of AD (Aloni et al., 2019).

A role of homeostatic synaptic plasticity in AD has been recently discussed (Jang and Chung, 2016; Styr and Slutsky, 2018). In the context of our data, which suggest a physiological role of Aβ, it can be speculated that the previously reported “synaptotoxic” effects of Aβ (e.g., Shankar et al., 2008; Mucke and Selkoe, 2012; Westmark, 2013; Wang et al., 2017; Zott et al., 2019) are the result of a pathologic overactivation of molecular machinery promoting synaptic homeostasis under physiological conditions. Consistent with this pathophysiological concept, Aβ-mediated “alterations” in LTP in AD models might reflect, at least in part, enhanced homeostatic synaptic plasticity, which rapidly returns potentiated synapses to baseline after LTP induction (Galanis and Vlachos, 2020). Clearly, the biological consequences of the “amyloidogenic/homeostatic” and the “non-amyloidogenic/Hebbian plasticity promoting” pathways warrant further investigation in the AD context, particularly because the amyloidogenic pathway is an important target for therapeutic intervention in AD (Yan and Vassar, 2014; Coimbra et al., 2018; Egan et al., 2019). Hence, some of the side effects observed in patients treated with β-secretase inhibitors (Coimbra et al., 2018; Egan et al., 2019) may have been caused by interference with the ability of healthy neurons to express homeostatic synaptic plasticity.
